# Effects of aging on the structural, mechanical, and thermal properties of the silicone rubber current transformer insulation bushing for a 500 kV substation

**DOI:** 10.1186/s40064-016-2549-y

**Published:** 2016-06-21

**Authors:** Zhigao Wang, Xinghai Zhang, Fangqiang Wang, Xinsheng Lan, Yiqian Zhou

**Affiliations:** State Grid Sichuan Electric Power Research Institute, Chengdu, 610072 China

**Keywords:** Silicone rubber, Polymers, Characterization, External insulation, Aging analysis

## Abstract

In order to analyze the cracking and aging reason of the silicone rubber current transformer (CT) insulation bushing used for 8 years from a 500 kV alternating current substation, characteristics including Fourier transform infrared (FTIR) spectroscopy, mechanical properties analysis, hardness, and thermo gravimetric analysis have been carried out. The FTIR results indicated that the external surface of the silicone rubber CT insulation bushing suffered from more serious aging than the internal part, fracture of side chain Si–C bond was much more than the backbone. Mechanical properties and thermal stability results illustrated that the main aging reasons were the breakage of side chain Si–C bond and the excessive cross-linking reaction of the backbone. This study can provide valuable basis for evaluating degradation mechanism and aging state of the silicone rubber insulation bushing in electric power field.

## Background

In recent years, the silicone rubber material has been widely used in the external insulation field of high voltage power transmission and transformation due to its excellent electrical insulation property, anti-pollution flashover performance, explosion-proof, hydrophobicity, and hydrophobicity transfer property (Amin and Salman 2006; Reynders et al. 1999; Liang et al. 2009; Papailiou and Schmuck 2013; Chen et al. 2015; Zhou et al. 2016). However, when it was used in Sichuan Power Grid of China, complicated environmental factors such as sun exposure, ultraviolet radiation, high temperature, high humidity, corona discharge, and high leakage current could accelerate the ageing process of the silicone rubber (Hackam 1999; Hillborg et al. 2001; Chandrasekar et al. 2007; Song et al. 2015). In many transformer substations from 110 to 500 kV used for years, some silicone rubber composite insulators of current transformer (CT), potential transformer (PT), circuit breaker, and surge arrester have showed different levels of ageing and cracking phenomena. It seriously affected safe operation of the power transmission and transformation equipments and electrical power system.

**Fig. 1:**
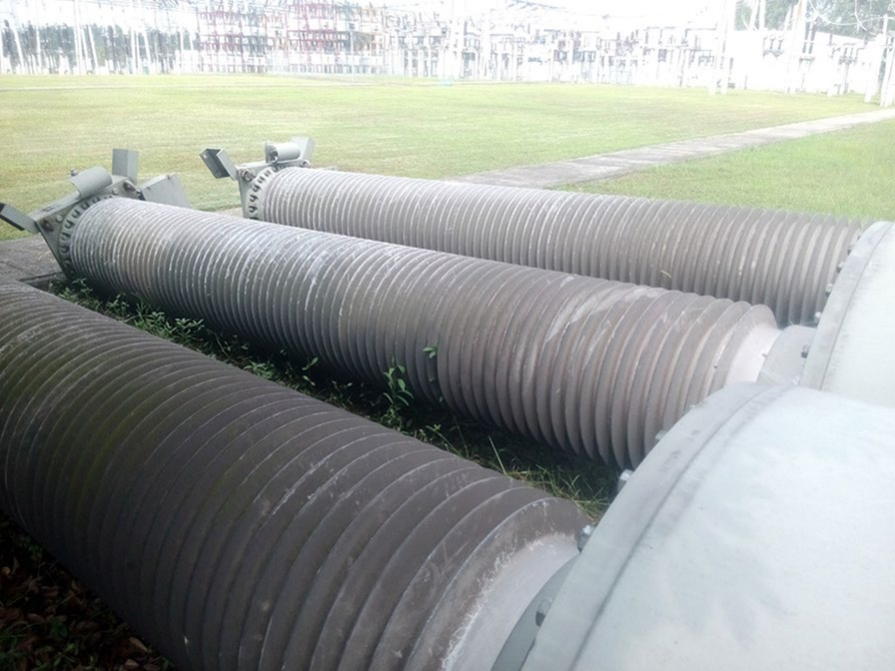
Picture of 500 kV CT composite insulators out of service

**Fig. 2:**
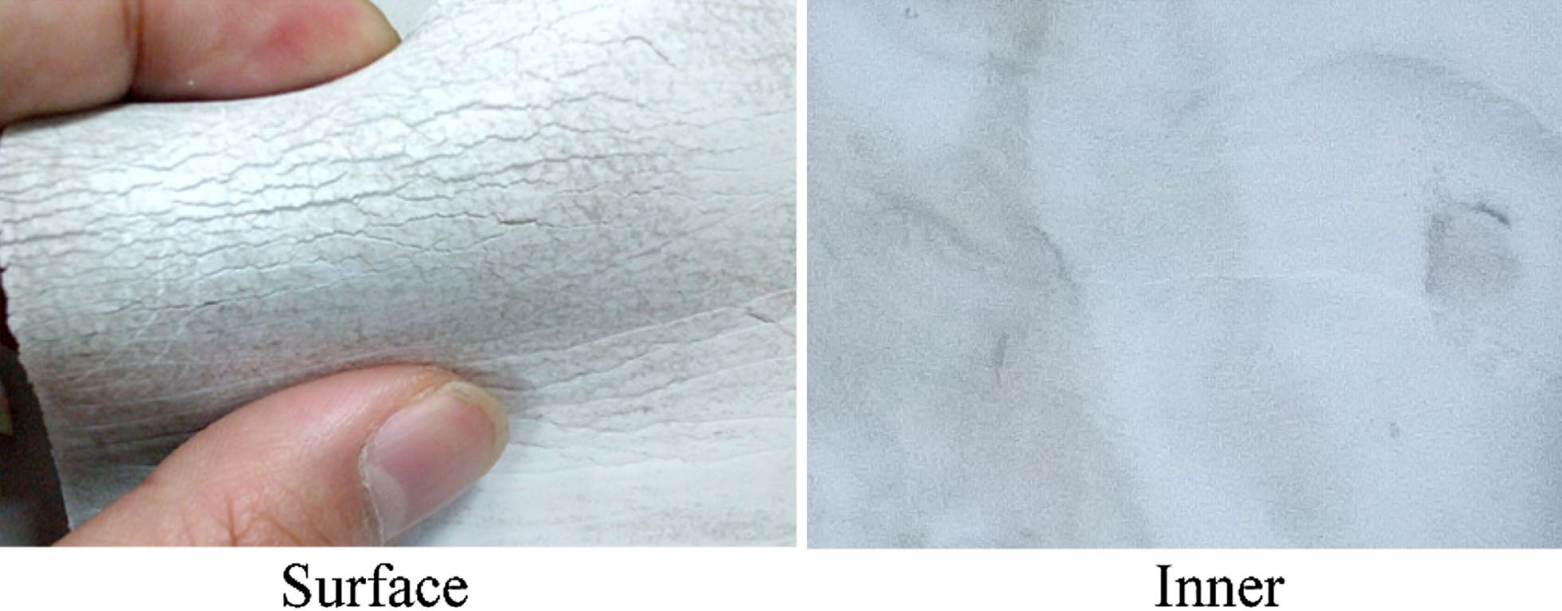
Cracking on the surface part (*left*) and intact inner part (*right*) of the CT insulation bushing

We have examined the composite insulators of CT (SAS550, MWB Shanghai Transformer Co., Ltd.) with 8 years service time from a 500 kV alternating current (AC) substation in Sichuan Power Grid of China. They were out of service now and the picture was given in Fig. 1. Cracking and chalking phenomena were observed on the surface of the insulation bushing of CT, while the inner parts remained intact, as shown in Fig. 2. This series of CT composite insulator was consisting of a silicone rubber bushing and a fiber-reinforced plastic (FRP) core. Although several researchers have paid attention to the composite insulator ageing, limited knowledge of the long-term performance of composite insulators at high voltage is yet available (Gubanski et al. 2007; Guo et al. 2010; Fernando and Gubanski 2010; Lutz et al. 2012; Jiang 2014). It is essential to investigate long-term ageing performance and ageing state assessment of the external insulation materials of silicone rubber with the help of chemical characterization methods.

In this paper, SAS550 CT insulation bushing (MWB Shanghai Transformer Co., Ltd.) used for 8 years from a 500 kV AC substation was investigated by means of several different test methods on the basis of previous work in our group (Wang et al. 2011a, b; Tan et al. 2013). Characteristics including Fourier transform infrared (FTIR) spectroscopy, mechanical property analysis, hardness, and thermo gravimetric analysis (TGA) have been carried out on the external surface and internal samples of the silicone rubber CT insulation bushing in different parts to study the change of molecular structure and ageing state.

## Experimental

### Materials

All the samples involved in this paper were collected from a SAS550 current transformer (MWB Shanghai Transformer Co., Ltd.) from a 500 kV AC substation which was manufactured in Dec. 2004 and have been in service for 8 years. The samples were collected from surface and inner of the silicone rubber insulation bushing, including the top part, middle part and bottom part, 6 types of samples were obtained: external surface of the bottom part, inner of the bottom part, external surface of the middle part, inner of the middle part, external surface of the top part, inner of the top part, they were named as S1, S2, S3, S4, S5, S6, respectively. All the samples were cut from on-site CT insulation bushings with the size of 30 mm × 30 mm × 2 mm. They were cleaned with anhydrous alcohol and deionized water to remove dust and were dried for 24 h at 50 °C in a vacuum oven. All the samples were collected in Aug. 2014 when these CTs had already been out of service.

### ATR-FTIR spectroscopy

Attenuated total reflectance Fourier transform infrared (ATR-FTIR) spectroscopy was utilized to analyze the change of the molecular structure and functional groups in surface and inner parts of the silicone rubber insulation bushing. Each specimen (2 mm in thickness) was pressed against the KBr plate directly. ATR-FTIR spectra of the silicone rubber CT insulation bushing samples were obtained on a Nicolet 560 FTIR spectrophotometer (Nicolet, USA) equipped with an attenuated total reflectance (ATR) attachment between 4000 and 500 cm^−1^ with a resolution of 2 cm^−1^.

### Mechanical properties

Mechanical properties were measured with an Instron 5567 (Instron, USA) universal electronic tensile machine. Dumbbell samples with dimensions of 25 mm × 6 mm × 2 mm were prepared for the tensile tests at a tensile rate of 500 mm/min according to GB/T 528-2009 (similar to ISO 37:2005) standard. The average result of five highest readings at peak load was reported as tensile strength. The strain values at the breaking point were used to obtain elongation at break (%). All mechanical values were taken from an average of five samples.

### Hardness

The Shore A hardness of all the samples were determined with a Shore Hardness Tester (Liuling LX-A, Shanghai, China) according to GB/T 531-2008 (similar to ASTM D2240).

### Thermo gravimetric analysis (TGA)

Thermal analysis of all the samples was performed with a TG 209F1 Iris (Netzsch, Germany) thermo gravimetric analyzer. 5–10 mg of test samples were cut from surface and inner parts of the silicone rubber insulation bushing in different locations. The samples were heated from 50 to 800 °C at a heat rate of 10 °C/min under a nitrogen atmosphere with a gas flow rate of 60 mL/min. The relative mass loss of the samples was recorded.

## Results and discussion

### ATR-FTIR

**Fig. 3:**
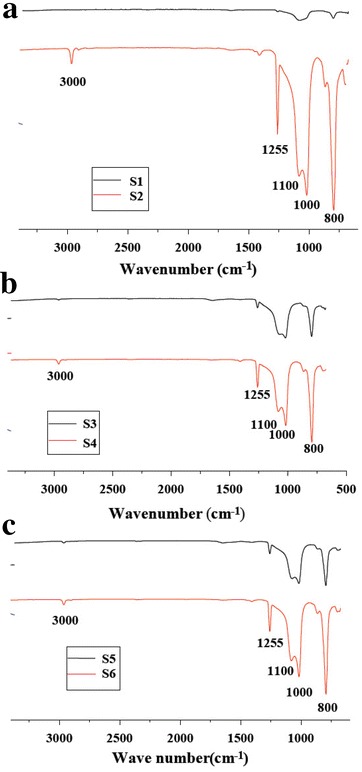
FTIR spectra of the silicone rubber CT insulation bushing samples: **a** S1 and S2, **b** S3 and S4, **c** S5 and S6

By the aid of attenuated total reflectance Fourier transform infrared (ATR-FTIR) characterization analysis of silicone rubber insulation bushing samples at different locations, the change of the molecular structure and functional groups can be analyzed, especially for the contrast between surface and inner of the material, thus the ageing extent can be judged. FTIR spectra of the silicone rubber CT insulation bushing samples are shown in Fig. 3. Seen from the spectra, the characteristic absorption peaks and the corresponding functional groups are as follows. The absorption peaks at 3000 cm^−1^ are corresponded to the stretching vibration of methyl (–CH_3_). The peaks at 1255 cm^−1^ are attributed to Si–CH_3_ bond. The strong absorption peaks at 1000–1100 cm^−1^ are ascribed to the presence of Si–O–Si. The strong stretching vibration peaks at 800 cm^−1^ demonstrate the existence of Si–(CH_3_)_2_. All these peaks are the characteristic peaks of silicone rubber.

In Fig. 3, all the absorption peaks at 3000 cm^−1^ (–CH_3_), 1255 cm^−1^ (Si–CH_3_), 1000–1100 cm^−1^ (Si–O–Si), and 800 cm^−1^ (Si–(CH_3_)_2_) of the external surface of the silicone rubber insulation bushing are much lower than those of internal ones, proving the reduction of Si–O and Si–C bond. In addition, the results reflect an important characteristic of the degradation of the silicone rubber insulation bushing: absorption peaks of Si–C bond decline much faster than absorption peaks of Si–O bond, which demonstrates that the fracture on the side-chain groups (mainly Si–(CH_3_)_2_) are much more than the backbone of the silicone rubber (Chen et al. 2015).

### Mechanical properties

Change in mechanical property is one of the most important performances on the material ageing behavior. As a kind of organic material, silicone rubber CT insulation bushings were exposed to the oxygen, sunlight, UV radiation, high temperature, rain, salt fog, corona discharge, and high leakage current, all the factors can accelerate the ageing process of the silicone rubber materials. Their mechanical performances will gradually decrease with the service time increasing, and the influence to the surface material is more significant. So tensile strength and elongation at break tests are required. The mechanical properties of the silicone rubber CT insulation bushing samples at different parts are listed in Table 1.

**Table 1:** Mechanical properties test results of the silicone rubber CT insulation bushing samples

Sample	Tensile strength (MPa)	Elongation at break (%)
S1	1.87	217
S2	2.88	267
S3	2.08	261
S4	2.55	256
S5	2.60	243
S6	2.66	236

As shown in Table 1, the tensile strength on the surface parts of the silicone rubber CT insulation bushing are significantly lower than those of internal parts. The change in the bottom part is the most obvious. In the case of the bottom part of the silicone rubber CT insulation bushing, the tensile strength decreases remarkably from 2.88 to 1.87 MPa when S2 (internal sample) compared with S1 (surface sample). Similarly, the tensile strength decreases from 2.55 to 2.08 MPa when S4 compared with S3, and the tensile strength decreases from 2.66 to 2.60 MPa when S6 compared with S5. The elongation at break of the silicone rubber CT insulation bushing sharply decreases from 267 to 217 % when the comparison is made between the internal sample (S2) and surface sample (S1). Whereas for S3 compared with S4, and S5 compared with S6, elongation at break values almost do not change. During the ageing course of polymer materials, chain scission, degradation, and cross-linking reactions may occur. In general case, tensile strength and elongation at break of polymer materials could decline after ageing. In this article, fracture and degradation of the side-chain groups and backbone of the silicone rubber result in reduction in tensile strength. When polymer materials are cross-linked, elongation at break will increase with the improvement of cross-linkage degree. If the degree of cross-linking continues to improve, excessive cross-linking will result in a decline in the elongation at break. The elongation at break of S1 is greatly reduced compared with S2, whereas S3 between S4 and S5 between S6 show little change, which illustrate that the degree of cross-linking of S1 is the maximum. Mechanical test results show that the surface parts of the silicone rubber CT insulation bushing have undergone serious ageing, ageing extent of S1 is the most severe. These results are in accordance with the FTIR results.

### Hardness

Shore hardness of the surface and inner of the silicone rubber CT insulation bushing were tested, the results are shown in Table 2. The hardness of the surface samples have a certain increase from the internal samples. Especially, the performance of external surface of the bottom part is very obvious. Comparison between S1 and S2 results shows that the degradation level of Sample S1 is much more serious than that of Sample S2, the Shore A hardness of S1 is about 33HA, much higher than that of S2 (25HA), which demonstrates that the cross-linking degree of S1 is relatively high. The hardness results have proven that the silicone rubber ageing phenomenon occurred mainly on the surface, the cross-linking degrees of the surface parts increased after ageing, along with hardness increased.

**Table 2:** Shore A hardness of the silicone rubber CT insulation bushing samples

Sample	S1	S2	S3	S4	S5	S6
Shore hardness (HA)	33	25	26	26	27	26

### Thermo gravimetric analysis

**Fig. 4:**
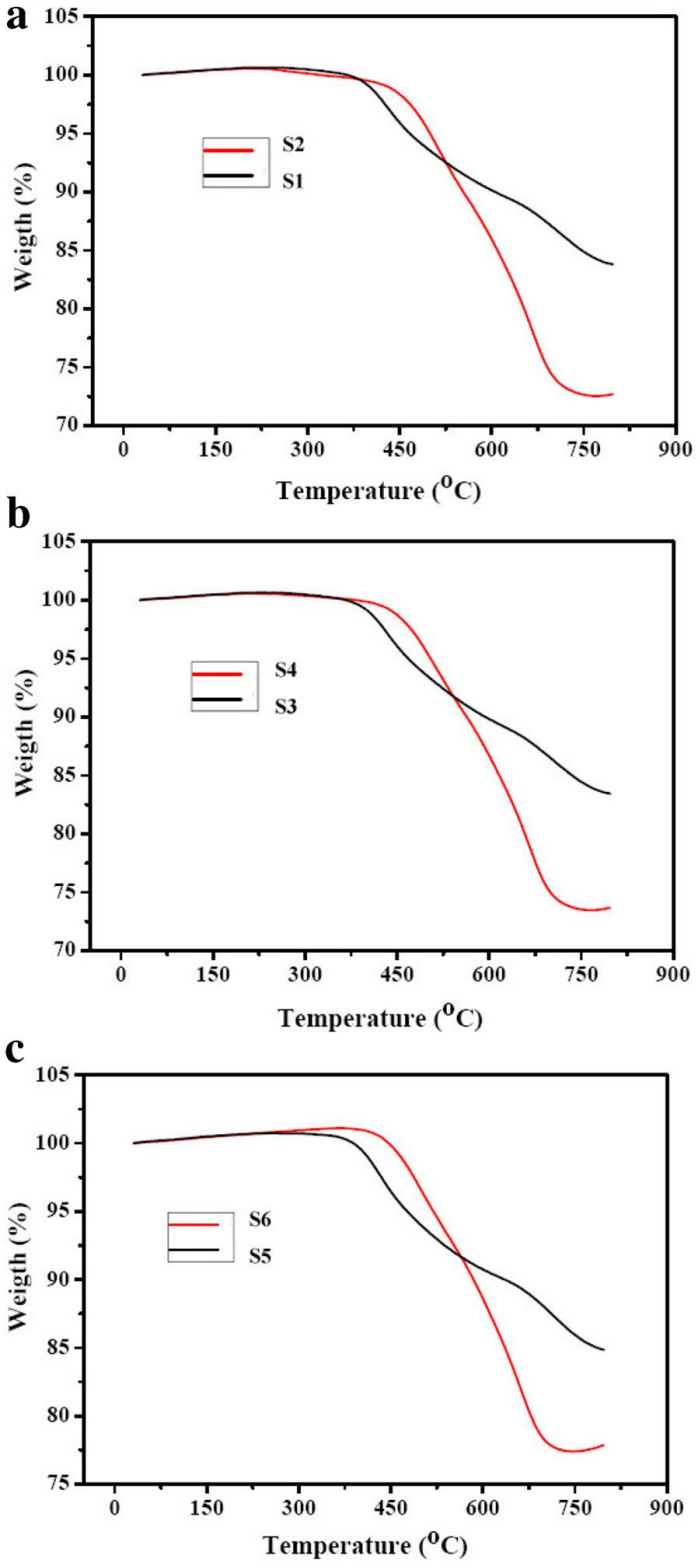
TG curves of the silicone rubber CT insulation bushing samples: **a** S1 and S2, **b** S3 and S4, **c** S5 and S6

Thermo gravimetric analysis (TGA) approach can effectively determine the thermal stability of the polymer materials. One of the most significant performances is the change in thermal decomposition temperature when the molecular structure changes. For the aged material, whose initial decomposition temperature tends to drop, the thermal stability is weakened. TG curves and data of the silicone rubber CT insulation bushing samples are shown in Fig. 4 and Table 3.

**Table 3:** Thermo gravimetric analysis data of the silicone rubber CT insulation bushing samples

Sample	T_onset_ (°C)	T_max_ (°C)	Residue at 800 °C (%)
S1	375.1	425.2	83.80
S2	427.2	665.2	72.70
S3	375.3	438.1	83.40
S4	427.6	664.1	73.47
S5	375.6	436.1	84.85
S6	428.1	664.4	77.89

As can be seen from the TG curves of the silicone rubber CT insulation bushing samples in Fig. 4, the thermal degradation of silicone rubber system takes two mass loss steps. The first step during the temperature range from 370 to 650 °C is attributed to the thermal decomposition of the side-chain groups of the silicone rubber. The other might be ascribed to the thermal degradation of the backbone during 650–800 °C. The decomposition process is characterized by the initial and maximum degradation temperatures, as summarized in Table 3. T_onset_ is the initial decomposition temperature, T_max_ is the temperature of the maximum rate of decomposition. Below 420 °C, inner parts of the silicone rubber have not exhibited weight loss, indicating that the molecular structure has not changed and ageing has not happened. For the surface parts of the silicone rubber, decomposition begins from 370 °C, it might be attributed to the thermal degradation of small molecules derived from side-chain groups. For instance, T_onset_ (375.1 °C) and T_max_ (425.2 °C) of S1 is lower than that of S2 (427.2, 665.2 °C respectively), indicating that surface parts of the silicone rubber have suffered to ageing worse than the internal samples. Table 3 also lists the residues at 800 °C, the values of the surface parts are higher than those of the inner parts. This is because surface parts of the silicone rubber CT insulation bushing have suffered to ageing due to sun exposure, ultraviolet radiation, corona discharge, and high leakage current in 8 years’ service duration. For the surface parts of the silicone rubber CT insulation bushing, a certain amount of organic materials had already been decomposed in long service time, they were lost as CO_2_ and H_2_O, remaining a large amount of inorganic filler materials in solid state and unchanged on heating. So the weight loss of surface parts in TG analysis should be smaller. Also TGA results are consistent with the results of mechanical properties and FTIR analysis.

## Conclusion

In this paper, the aging performances of the silicone rubber CT insulation bushing which has been in service for 8 years from a 500 kV AC substation were analyzed by FTIR, mechanical experiments, hardness, and TGA. The results show that the external surface of the silicone rubber CT insulation bushing has seriously aged, the molecular structure changed, strength declined, surface hardness increased, and thermo stability declined. This study can provide valuable basis for evaluating aging mechanism and aging degree of the silicone rubber insulation bushing in electric power field.
